# Emerging Biomarkers in Cardiac Sarcoidosis and Other Inflammatory Cardiomyopathies

**DOI:** 10.1007/s11897-024-00683-9

**Published:** 2024-10-04

**Authors:** Joseph El Roumi, Ziad Taimeh

**Affiliations:** Section of Heart Failure and Transplantation Medicine; Robert and Suzanne Tomsich Department of Cardiovascular Medicine, Miller Family Heart, Vascular & Thoracic Institute; Cleveland Clinic, 9500 Euclid Ave, J3-4, Cleveland, OH 44195 USA

**Keywords:** Cardiac sarcoidosis, Inflammatory cardiomyopathy, Myocarditis, Biomarkers

## Abstract

**Purpose of Review:**

Cardiac sarcoidosis and other inflammatory cardiomyopathies are disorders causing cardiac inflammation and leading to heart failure, arrythmias and cardiac arrest. Diagnosis of these entities remains challenging and multimodal. Thus, there is a growing need to develop reliable biomarkers that can aid in the diagnosis. This review aims to summarize and highlight recent findings in the field of biomarkers for cardiac sarcoidosis and inflammatory cardiomyopathy.

**Recent Findings:**

Multiple categories of biomarkers including novel molecules are being investigated with the latest evidence showing promising results. Some of these biomarkers are proven to be useful as diagnostic and prognostic aids in cardiac sarcoid and inflammatory cardiomyopathy.

**Summary:**

The identification of cost-effective and accurate biomarkers is useful not only for enhancing diagnostic accuracy but also for informing therapeutic decision-making processes. This advancement would facilitate the timely institution of immunosuppressive therapies, ultimately leading to improved patient outcomes.

## Introduction

Sarcoidosis is a systemic inflammatory autoimmune disorder of unknown etiology characterized by the presence of non-caseating granulomas [1]. Cardiac involvement is associated with worse prognosis and carries an elevated risk of heart failure (HF), ventricular arrythmias and sudden death [2]. The prevalence of cardiac sarcoidosis (CS) on autopsy studies is reported as 20–27% in the United States [3–5], compared to 58% in Japan [6]. This disease remains a diagnostic challenge with no reliable biomarker for establishing the diagnosis or monitoring response to therapy [1]. Endomyocardial biopsy (EMB), albeit an invasive procedure with low sensitivity due to the patchy nature of the disease, is considered the gold standard for diagnosing CS [7, 8]. Recent advances in cardiac multimodality imaging (CMMI) have allowed identification of myocardial inflammation and disease activity in CS by using 18F-fluorodeoxyglucose positron emission tomography (FDG-PET) and identification of areas of fibrosis and inflammation by late gadolinium enhancement (LGE) on cardiac MRI [9]. Although none of these findings are specific for CS, but in the right medical context and with high clinical suspicion, the diagnosis of CS can be made according to Heart Rhythm Society and Japanese Circulation Society criteria [1, 10].

In the spectrum of inflammation, myocarditis refers to a heterogeneous group of disorders characterized by acute myocardial inflammation [11, 12]. Myocarditis can present acutely, sub-acutely, or chronically, with symptoms ranging from mild chest pain, fatigue, and palpitations to severe heart failure or sudden cardiac death. Its fulminant form is one of the main causes of cardiogenic shock and death in young adults [13–15]. It is mainly associated with infectious etiologies (viral, bacterial, fungal and protozoan) but can also be triggered by other non-infectious etiologies (immunological syndromes, hypersensitivity, toxins and substances) [16, 17]. Myocardial inflammation is intricately regulated by a plethora of immune cells, cytokines, and autoantibodies, wherein the innate immune system is the primary catalyst for myocardial injury during the acute phase, while its acquired immune counterpart dominates in the chronic remodeling phase [16, 18]. Although it might not be inherently different from myocarditis, inflammatory cardiomyopathy (IC) is used to describe the progression of myocarditis into a chronic inflammatory phase that causes further cardiac remodeling, permanent myocardial fibrosis, and ultimately leads to a dilated cardiomyopathy phenotype [17, 19].

Since there is significant overlap between CS and IC, both causing cardiac inflammation and scar formation on CMMI, establishing an accurate diagnosis of IC can be problematic. Moreover, prompt identification of both these conditions is imperative to institute timely immunosuppressive therapies and forestall the development of HF, arrhythmias, and conduction abnormalities [1, 17]. EMB is of limited utility in the diagnosis of myocarditis and IC and is only reserved to suspected patients who endorse high risk features (cardiogenic shock, high-degree atrioventricular block, malignant ventricular arrythmias) [12]. Despite advances in the world of cardiac imaging and in the search for high-sensitivity biomarkers, there remains multiple gaps in the diagnosis and treatment of CS and IC. There is a need to develop accurate biomarkers indicative of disease progression, aimed at risk stratification in affected patients, thus providing insight for diagnostic and therapeutic decision-making [20].

From this perspective, the objective of our review is to elucidate the research endeavors undertaken over the preceding decade aimed at identifying biomarkers with clinical utility for serving as diagnostic aids and for monitoring disease activity and treatment response in CS and IC [17, 20].

## Role of Emerging Biomarkers

According to the Biomarkers and Surrogate End Point Working Group in 2001, a biomarker is defined as “a characteristic that is objectively measured and evaluated as an indicator of normal biological processes, pathogenic processes, or pharmacologic responses to a therapeutic intervention” [21]. Hence, in contemporary clinical practice, the array of measured biomarkers should encompass samples from blood, bodily fluids, and tissues, with the aim of enhancing comprehension of pathological conditions such as CS and myocarditis. Furthermore, these biomarkers should exhibit reliability and minimal invasiveness, coupled with sufficient sensitivity and specificity, while remaining cost-effective and capable of prognostic determination [20]. The pursuit of an ideal biomarker is protracted and arduous, yet the biomarkers highlighted in this article pertaining to CS and myocarditis epitomize the most extensively researched and clinically pertinent ones within the scope of current medical knowledge.

## Part 1: Biomarkers in CS

Table 1 represents pivotal biomarkers alongside key studies elucidating their efficacy and clinical utility in the context of CS

**Table 1:** Summary of biomarkers in cardiac sarcoidosis

Biomarker	Cohort Reported	Total Number of Patients Enrolled	Summary of Study Endpoints and Results	Biomarker Utility
ACE	Kiko, et al. 2018 [25]	172	Higher levels associated with SS but not isolated CS (*P*=0.041)	Diagnosis
	Kolluri, et al. 8	232	Presence of LGE on CMR (mean difference, 14.7; *P*=0.03)	Prognosis
Soluble interleukin-2 receptors	Kobayashi, et al. 2021 [30]	83	Arrythmias, all-cause mortality and HF hospitalizations (*P*=0.037)	Prognosis
High-sensitivity Troponin T	Kandolin, et al. 2015 [31]	50	Associated with LV dysfunction at presentation, signficiant decrease in serum level after 4 weeks of steroid therapy (*P*=0.0003)	Disease activity and treatment response
	Kolluri, et al. 8	232	Higher risk for LVAD implantation, heart transplant, death (HR= 1.06, *P*=0.006)	Prognosis
High-sensitivity Troponin I	Kandolin, et al. 2015 [31]	12	Associated with LV dysfunction at presentation, signficiant decrease in serum level after 4 weeks of steroid therapy (*P*=0.043)	Disease activity and treatment response
	Kiko, et al. 2018 [25]	172	Predictor of fatal arrythmias (HR= 2.418, *P*=0.003)	Prognosis
BNP	Miyakuni, et al. 2022 [37]	238	HR= 2.06 [95% CI, 1.19–3.55]; *P*=0.010	Prognosis
	Kiko, et al. 2018 [25]	172	Predictor of heart failure (AUC= 0.85, *P*=0.002)	Diagnosis and prognosis
NT-pro BNP	Kolluri, et al. 8	232	Higher risk for LVAD implantation, heart transplant, death (HR=1.31, *P*< 0.001)	Prognosis
1,25 dihydroxyvitamin D (1,25-OHVit-D)	Kolluri, et al. 8	232	Uptake on cardiac FDG-PET (AUC=0.8, *P*=0.03 )	Diagnosis and disease activity
Urinary 8-hydroxy-2'-deoxyguanosine	Kobayashi. et al. 2015 [30]	89	Correlates with inflammatory activity of CS as evident on PET-CT(AUC, 0.98; 95% CI, 0.94–1.02; optimal cutoff value 13.1 ng/mg creatinine)	Prognosis and disease activity
	Myoren, et al. 2016 [44]	30	Urinary level ≥19.1 ng/mg·Cr predictor of cardiovascular death (*P* = 0.001)	Prognosis and treatment response
	Ishiguchi, et al. 2017 [43]	62	VT in patients with active CS (HR=0.61, *P*<0.00001)	Prognosis
miRNA-126	Fujiwara, et al. 2018 [49]	27	No significant differences between isolated CS and SS with cardiac involvement	Diagnosis of CS in patients with HF
miRNA-223	Fujiwara, et al. 2018 [49]	27	No significant differences between isolated CS and SS with cardiac involvement	Diagnosis of CS in patients with HF

## Angiotensin-Converting Enzyme (ACE) and Soluble Interleukin-2 Receptor (sIL-2R)

Serum angiotensin-converting enzyme (ACE) stands as the most studied biomarker in sarcoidosis, with earlier investigations suggesting elevated ACE levels in afflicted patients. It has also been proposed that ACE might serve as a diagnostic tool and a gauge for disease activity in sarcoidosis, since it is secreted from activated macrophages within sarcoid granulomas [22, 23]. Nonetheless, the clinical applicability of ACE measurement remains dubious, given its propensity for elevation across diverse granulomatous conditions, encompassing sarcoidosis, tuberculosis, and fungal infections [22, 24].

The utility of ACE in CS was investigated in a cohort of 172 patients, showing that ACE levels were significantly higher in systemic sarcoidosis (SS) than in isolated CS (ACE, 25.8 versus 18.2 U/l, *P* = 0.041). In addition, serum ACE was not able to predict fatal arrhythmias and HF, even after adjusting for LV ejection fraction (LVEF) [25].

In a 2021 study conducted at the Mayo Clinic involving a cohort of over 230 patients diagnosed with CS, it was demonstrated that serum ACE levels were predictive for the presence of LGE on cardiac MR [8]. Specifically, at an optimized threshold of 34 U/L, the sensitivity and specificity for detecting LGE on CMR were determined to be 55.3% (95% CI, 40.1%-69.8%) and 91.7% (95% CI, 61.5%-99.8%), respectively [8]. The identification of LGE on CMR has significant implications, correlating with adverse clinical outcomes such as mortality and the occurrence of ventricular arrhythmias, as well as resistance to corticosteroid therapy in CS [26, 27]. Although the diagnostic utility of ACE in CS remains circumscribed, its prognostic value in this context is noteworthy. Therefore, the consideration of measuring serum ACE levels in patients undergoing evaluation for CS seems warranted, with elevated levels prompting further assessment via cardiac MRI.

SIL-2R, a proposed marker of T-cell activation and disease activity in sarcoidosis, was previously proven to be superior to other biomarkers in terms of screening for SS and determining the severity of lung lesions in affected patients [28, 29]. In a retrospective study by Kobayashi on individuals who were admitted for laboratory and imaging investigations for suspected CS, elevated sIL-2R levels was shown to positively correlate with worse clinical outcomes (arrythmias, all-cause death and HF hospitalizations; *P* = 0.037) even after adjustments for age, BNP, estimated GFR, cardiac metabolic volume (CMV), LVEF, and LGE [30]. However, this biomarker did not correlate with isolated uptake on cardiac PET, and showed an association with lymph node uptake, thus reflecting systemic inflammation [30]. A similar result was also described by the Mayo Clinic group, where sIL-2R were found to be significantly higher in non-isolated CS than in isolated CS (1170 versus 380.5 pg/mL, *P* = 0.038) [25]. Based on these findings, sIL-2R has a limited role in the diagnosis of CS but could be a promising marker for prognosis and adverse events in those already diagnosed with this disorder.

## Cardiac Troponins (T/I)

Cardiac troponins, recognized as hallmark indicators of myocardial injury emanating from damaged cardiomyocytes [22], have garnered attention in the context of cardiac sarcoidosis (CS). Several investigations have associated elevated levels of cardiac troponins in CS with disease activity assessed via FDG-PET, suggesting a potential prognostic significance in this setting [22–24]. Notably, in 2015, Kandolin et al. demonstrated a correlation between elevated cardiac troponin I and T levels in CS patients and left ventricular dysfunction at presentation, with subsequent normalization following steroid treatment indicating a favorable prognosis [31]. Furthermore, while troponin I elevation was observed in both SS and CS [19], Kiko et al. revealed its association with fatal ventricular arrhythmias in the CS group, even after adjusting for left ventricular ejection fraction (LVEF) [19]. Conversely, the group from Mayo Clinic found no significant correlation between cardiac troponins and LVEF at presentation yet identified an association between troponin T elevation and adverse outcomes such as the need for advanced therapies (AT) and mortality (hazard ratio 1.06, *P* = 0.0006) [4]. Thus, serum cardiac troponins emerge as promising markers for prognostication and assessment of treatment response in CS.

## Brain Natriuretic Peptides (BNP and NT-pro BNP)

Natriuretic peptides have been widely used to establish the diagnosis of HF with higher serum concentrations correlating with a greater risk for adverse short- and long-term outcomes in HF, including all-cause and cardiovascular death [32]. On one hand, multiple reports have demonstrated the utility of BNP to be useful in detecting cardiac involvement in patients with SS [33, 34]. At a cutoff value used for discrimination at 40 pg/mL, BNP outperformed other biomarkers such as ACE and sILR2, with an AUC of 0.85 (*p* = 0.002) at predicting HF occurrence [25]. However, interpreting serum BNP can be challenging, since multiple factors such as left ventricular hypertrophy, atrial fibrillation, older age and impaired renal function may lead to serum elevations in this biomarker [35, 36]. Moreover, in the post-hoc analysis of the ILLUMINATE‐CS (238 patients, median follow-up 3 years), high baseline BNP was described as an independent predictor of the combined end point of all‐cause death, heart failure hospitalization, and fatal ventricular arrhythmia (log‐rank *P* = 0.004) in patients with CS without heart failure at the time of diagnosis, and these findings were also retained even after adjusting for age, kidney function and baseline LVEF (HR, 2.06 [95% CI, 1.19–3.55]; *P* = 0.010) [37]. On another hand, NT-pro BNP was found to be significantly elevated in patients with definite CS, probably due to their advanced myocardial disease and worse hemodynamics [8]. Dichotomized at a cutoff of 700 pg/ml (*P* = 0.009), this biomarker was associated with the need for AT, mortality and death, irrespective of baseline LVEF [8]. Considering these findings, natriuretic peptides emanate as important diagnostic and prognostic biomarkers for CS.

## Dihydroxy Vitamin D (1,25-OHVit-D)

The earliest description of elevated circulating 1,25-(OH) vit-D and perturbations in calcium metabolism in sarcoidosis dates to 1979 [30]. Initially, this observation was attributed to augmented vitamin D synthesis and reduced degradation, coupled with enhanced intestinal calcium absorption, culminating in hypercalcemia [31–34]. Subsequent in vitro investigations utilizing cultured pulmonary alveolar macrophages and sarcoid lymph node homogenates have delineated the extrarenal conversion of 25-(OH) vitamin D to 1,25-(OH) vitamin D through heightened expression and activity of 1-α hydroxylase in individuals with sarcoidosis [35–37]. Moreover, it has been elucidated that monocytes, dendritic cells (DCs), macrophages, B-cells, and T-cells exhibit localized 1-α hydroxylase activity [38–42]. This immunomodulatory function plays a key role in the significance in the pathogenesis of granulomatous disorders, particularly in the context of sarcoidosis [43, 44]. Serum elevations in 1,25-(OH) vit-D have been positively correlated with disease activity in SS [38], but very few studies have explored the role of 1,25-(OH) vit-D as a potential biomarker in CS. Encompassing 232 patients, the study from Mayo Clinic has avidly reported in 2022 a positive correlation between 1,25-(OH) vit-D and the presence of uptake on cardiac FDG-PET CT scan with a sensitivity of 55% and specificity of 100% at an optimal threshold of 66 pg/mL [8]. This finding may be reflective of advanced granulomatous burden in the heart, leading to higher degree of inflammation and disease activity which may portend worse prognosis in afflicted patients [8].

## Urinary 8-hydroxy-2'-deoxyguanosine (8-OHdG)

8-OHdG, an oxidized nucleoside of DNA, has been previously found to be highly upregulated in diseased cardiac states such as dilated cardiomyopathy and previous myocardial infarction. During the active phase of CS, it has been hypothesized that enhanced production of reactive oxygen species leads to nucleic and mitochondrial DNA oxidization, resulting in urinary excretion of 8-OHdG, a marker of high oxidative stress [39–42]. Three notable studies conducted in Japan have sought to elucidate the diagnostic and prognostic significance of 8-OHdG as a biomarker in CS patients [43–45]. In 2015, Kobayashi et.al demonstrated using immunohistochemical staining, antibodies targeting 8-OHdG in a subset of patients with CS compared to controls, who exhibited no such staining. Moreover, increased urinary concentrations of 8-OHdG were increasingly found in blood samples from active CS patients when compared to those with quiescent disease and dilated cardiomyopathy (DCM). This cohort also revealed that urinary 8-OHdG increased in proportion to the inflammatory activity of CS thus correlating with disease activity (AUC: 0.98; 95% CI,0.94–1.02) with a cutoff value of 13.1 ng/mg Cr, a sensitivity of 88.2% and a specificity of 92.9%. Additionally, diminished urinary excretion of 8-OHdG was associated with a favorable response to corticosteroid therapy, as shown by reduced uptake on FDG-PET (n = 26, r = 0.59, *P* = 0.01) [45].

Myoren and colleagues aimed to further characterize urinary 8-OHdG as a prognosticator of cardiovascular (CV)-related death in 30 patients with CS. By performing univariate and multivariate analyses, this biomarker outperformed BNP in predicting CV-related mortality in corticosteroid-treated subjects with CS, thereby identifying a subgroup potentially resistant to treatment (urinary concentration > 19.1 ng/mg·Cr with *P* = 0.001) [44]. Moreover, in their 2017 study, Ishiguchi et. al eloquently demonstrated that U-8-OHdG as an independent determinant for VT occurrence in those with active CS as demonstrated by FDG-PET, with a cutoff level of ≥ 17.5 ng/mg·Cr, a sensitivity of 89% and specificity of 83%, and area under the curve of 0.90. Furthermore, U-8-OHdG levels decreased in parallel with the number of VT episodes before and after corticosteroid therapy [43]. All these findings demonstrate that urinary 8-OHdG may correlate with disease activity in CS, play a role as a gauge to monitor treatment response and resistance, and may predict CV-related death and ventricular tachycardia (VT) occurrence.

## Micro RNAs-126 and 223

MicroRNAs (miRNAs), short single-stranded RNA molecules typically comprising 21–24 nucleotides, are considered as key modulators of genetic expression [46]. Previous investigations have highlighted their potential dysregulation in the context of cancer and cardiovascular diseases, thereby influencing the course of these conditions [46–48]. Since circulating miRNAs in peripheral blood have shown great promise as novel biomarkers for cardiovascular disease, we refer to one of the seminal studies conducted by Fujiwara et.al in 2018 aimed at elucidating their diagnostic utility in the diagnosis of CS in patients with HF [49].Although the study's cohort was relatively modest, findings revealed significant upregulation of miRNA-126 and miRNA-223 (*P* < 0.0001 and *P* = 0.0002, respectively) in the serum of individuals with CS and HF compared to control subjects. Importantly, this aberrant expression did not differ significantly between those with isolated CS and those with concurrent extracardiac manifestations. Furthermore, these miRNA levels did not correlate with the severity of HF, as shown by their lack of association with NT-pro BNP levels or LV dimensions assessed by echocardiography. In summary, miRNAs exhibit a promising role as diagnostic adjuncts in the realm of CS; however, further dedicated investigations with larger sample sizes and longitudinal blood sampling are warranted to comprehensively delineate their role as "next-generation biomarkers" in this clinical context [49].

## Cell-free DNA (cfDNA)

When a cell undergoes necrosis or apoptosis, fragments of genomic DNA, also called cfDNA, are released into the bloodstream. Over the past few years, this biomarker has emerged as a promising tool in the monitoring of rejection in orthotopic heart transplant (OHT) recipients [50]. A pilot study is currently being conducted to investigate the utility of this biomarker in identifying granulomatous myocarditis in patients with sarcoidosis [51].

## Part 2: Biomarkers in IC

The role of the following biomarkers studied in the realm of IC will be described according to the major subtype and/ or causative agent.

## Lymphocytic Myocarditis

Macrophages, T lymphocytes and eosinophils are the immune mediators implicated in the immune dysregulation of myocarditis and IC. Since this group of diseases has unique inflammatory cytokine upregulation and immune cell expression throughout its stages, identification of select immune biomarkers can aid in early diagnosis and patient risk stratification [52, 53].

Heparin binding proteins (HPBs), secreted by neutrophils, are one the first players involved in the initial inflammatory response [54]. More than 435 HPB genes were isolated so far, and they mainly function by facilitating leukocyte extravasation and migration to the site of infection, in addition to amplifying the immune system response by activating other immune cells such as macrophages and monocytes [54]. Although they are sensitive biomarkers for the detection of myocardial inflammation, they lack specificity but can serve as diagnostic adjuncts to other markers used in combination [20, 54].

Serum alarmins S100A8 and S100A9 are proinflammatory molecules, mainly expressed by monocytes and neutrophils in the initial phase of myocarditis. In 2020, Muller et.al published their study on serum alarmins in a cohort of 227 patients and found that elevations in these biomarkers were significantly associated with disease activity compared to controls. These biomarkers displayed a sensitivity of 90.6% and a specificity of 92% in the diagnosis of acute lymphocytic myocarditis [55].

## Immune Checkpoint Inhibitor (ICI)-Related Myocarditis

Troponin I spillover observed during cardiac damage is usually detected by troponin assays, such as conditions causing ischemic myocardial damage or in direct immune-mediated myocardial injury. Although only one third of patients with suspected myocarditis have serum troponin I elevation [56], routine use of this biomarker is endorsed by the European Society of Cardiology in patients with suspected myocarditis. Despite its limited sensitivity and specificity [56, 57], the role of troponin I has shown tremendous utility in the detection and monitoring of ICI-related myocarditis, a rare condition (incidence 0.04% to 1.14%) that is associated with high mortality rates ranging between 25 and 50% [58]. Conversely, serum troponin elevation was found to be elevated in 94% of patients with ICI-related myocarditis and was found to be prognostic in that setting, with serum values > 1.5 ng/mL associated with a fourfold increase in major adverse effects (MACE) defined as cardiovascular death, cardiogenic shock, cardiac arrest, or complete heart block [59]. Based on these findings, serum troponin I was incorporated in risk stratification and surveillance of high-risk patients undergoing cancer treatment with an ICI [59, 60]. In contrast to troponin I, serum natriuretic peptides were not predictive of MACE, although they were elevated in 66% of patients with ICI-related myocarditis, probably due to the development of HF and LV dysfunction [59]. From this standpoint, obtaining serum natriuretic peptides is reasonable once HF is suspected, but values within the reference range should preclude the diagnosis of ICI-related myocarditis [61].

## Fulminant Myocarditis

As previously coined, miRNAs are increasingly recognized as promising tools in CV diseases and thanks to the advent of efficient and affordable genetic sequencing tools, miRNAs are now easily obtained from profile sequencing [20]. Fulminant myocarditis (FM) is an acute and rapidly progressive inflammatory illness characterized by arrythmias and hemodynamic perturbations leading to significantly elevated mortality and need for OHT. The human miRNA miR-4763-3p was found to be significantly upregulated in adult FM with high sensitivity and specificity, but not in the setting of myocardial infarction [62]. Moreover, this miRNA may be useful in predicting prognosis and disease severity since it plays a role in genetic silencing of inflammatory and cardiac injury response pathways in FM [62]. The human counterpart of the murine miRNA mmu-miR-721, hsa-miR-Chr8:96, was able to demonstrate selective upregulation across four distinct cohorts of human adult FM. Statistical analyses revealed a receiver operating characteristic curve of 0.927, indicative of the discriminatory potential of this miRNA in distinguishing this entity from myocardial infarction [63]. In a study by Zhang et.al in 2021 comprising 236 patients, hsa-miR-30a, hsa-miR-192, hsa-miR-146a, hsa-miR-155, and hsa-miR-320a were validated as significantly and differentially expressed in adult FM (*p* < 0.05), thus serving as potential biomarkers in the diagnosis of this condition [64]. Conversely, using a multivariate logistic regression model, hsa-miR-155 and hsa-miR-320a demonstrated high precision in the diagnosis of FM with an AUC of 0.944 and was able to accurately distinguish it from its non-fulminant counterpart [64]. It was also found that in patients with FM, a rise in serum troponins correlates with impairment and reduced recovery rates of cardiac function. Moreover, serum troponin elevation was linked to higher in-hospital mortality and worse prognosis, despite the use the mechanical circulatory support [65]. Finally, in a study conducted in Wuhan, China, on a cohort of FM, soluble ST2 (sST2) showed marked dynamic changes from disease onset to resolution [66]. Moreover, this biomarker was able to differentiate FM from non-FM or other FM-unrelated acute HF more robustly than N-terminal pro-B-type natriuretic peptide or cardiac troponin I with an optimal cutoff value of 58.39 ng/mL, a sensitivity of 85.7% and specificity of 94.7% [66].

## Autoimmune Myocarditis (AM)

Autoimmune myocarditis can arise independently or in association with systemic autoimmune disorders such as systemic lupus erythematosus, rheumatoid arthritis, or Sjögren's syndrome [67]. It is primarily driven by the adaptive immune system which signals circulating B cells to produce autoantibodies that are directed against myocardial structures (β1-adrenergic receptors, myosin heavy chain, cardiac troponin, Na/K ATPase) [68]. In a cohort of 111 patients with HF and suspected autoimmune myocarditis, immunoglobulin free light chains were found to be a valuable tool (AUC = 0.87) in the diagnosis of this disorder with further compelling evidence revealing that a low kappa/lambda ratio may serve as a prognostic indicator of favorable survival outcomes in this clinical context [69].

## Giant Cell Myocarditis (GCM)

GCM is a rapidly progressive and potentially fatal necrotizing subtype of myocarditis that affects young and middle-aged adults. It is characterized by T-lymphocyte mediated myocardial damage and inflammation (multinucleated giant cells seen on histopathology), leading to severe HF and cardiogenic shock. Diagnosis of this condition relies on high clinical suspicion, which may prompt the treating cardiologist to pursue an EMB for accurate diagnosis and to initiate immunosuppressive therapy [70]. Serum troponin I elevation is rather disappointing in the setting of GCM, with studies showing no link between serum values and histologic severity of myocyte necrosis, with some reports showing undetectable troponin I in the setting of substantial myocardial necrosis seen on EMB [17, 70, 71]. Interestingly, troponin T has demonstrated a promising role in risk stratification, with elevated values being associated to with a reduced transplant-free survival rate [17]. Moreover, Ekström et.al observed that elevated troponin T at presentation in patients with GCM were linked to higher mortality rates and the need for OHT [72].

## Eosinophilic Myocarditis (EM)

EM is an underrecognized and rare entity usually diagnosed at post-mortem examination [73]. On histopathology, it is mainly characterized by myocardial inflammation and injury due to an eosinophil-predominant inflammatory infiltrate leading to LV dysfunction [57]. In a case series of 29 patients with biopsy-proven EM, more than 50% of cases presented with a fulminant and necrotizing disease that can lead to cardiogenic shock and death [74]. In rare occasions, EM may lead to a chronic form of restrictive cardiomyopathy also known as Loeffler cardiomyopathy [75]. This entity is caused by hypersensitivity reactions [76], immune-mediated disorders, such as eosinophilic granulomatosis with polyangiitis (EGPA), undefined complex hypereosinophilic syndrome (HES) or its myeloproliferative variant [77], infections and cancer [75]. However, in most cases, the etiology remains unknown. Although part of the diagnostic criteria for EM [78], peripheral eosinophilia can be absent in 25% of cases, making the diagnosis without an EMB very difficult [75, 79]. In only 12.8% cases, peripheral eosinophilia may develop between the second and sixth day of presentation [75]. Despite not being a novel biomarker, it is worth mentioning the eosinophil cationic protein (ECP) which is released after the degranulation of eosinophils [80]. This biomarker was found to be positively associated with disease activity and can be used as gauge for treatment response since lower levels of this protein were correlated with clinical improvement [81].

## Conclusion

The past few years have witnessed tremendous advances in the field of biomarker research, aimed at identifying biomarkers able to aid in the diagnosis and prognostication of CS and myocarditis. Robust biomarkers not only should stand the test of time and provide clinicians with adequate sensitivity and specificity but should also be reliable and easy to perform. The rise of novel miRNAs is the latest in the field and is worth highlighting, as they seem to offer promising value as more dedicated research is undertaken, while other traditional biomarkers such as serum ACE and troponin seem to exhibit more clinical usefulness than previously thought.

## Key References

- Kolluri, N., et al., *Routine Laboratory Biomarkers As Prognostic Indicators of Cardiac Sarcoidosis Outcomes.* Sarcoidosis Vasc Diffuse Lung Dis, 2022. **39**(3): p. e2022023.
  - **This paper elucidates the important roles of biomarkers such as troponin, pro-BNP and ACE in a large cohort of patients with cardiac sarcoidosis**
- Suresh, A., P. Martens, and W.H.W. Tang, *Biomarkers for Myocarditis and Inflammatory Cardiomyopathy.* Curr Heart Fail Rep, 2022. **19**(5): p. 346–355.
  - **A recent review that summarizes new molecular biomarkersand their respective roles in the field of myocarditis and inflammatory cardiomyopathy.**
- Wang, J., et al., *Soluble ST2 Is a Sensitive and Specific Biomarker for Fulminant Myocarditis.* J Am Heart Assoc, 2022. **11**(7): p. e024417.
  - This study reveals the latest evidence behind the promising role of a novel biomarker, soluble ST 2, in the diagnosis of fulminant myocarditis and prognostic role in HF development in affected patients.
- Palaskas, N., et al., *Immune Checkpoint Inhibitor Myocarditis: Pathophysiological Characteristics, Diagnosis, and Treatment.* J Am Heart Assoc, 2020. **9**(2): p. e013757.
  - **With recent advances in cancer therapies, this article provides a comprehensive summary of immune checpoint inhibitor myocarditis, an entity that is currently recognized as a life-threatenting form of drug-induced myocarditis, requiring prompt diagnosis and early treatment.**

## Data Availability

No datasets were generated or analysed during the current study.

## References

[1] Trivieri MG, et al. Challenges in Cardiac and Pulmonary Sarcoidosis: JACC State-of-the-Art Review. J Am Coll Cardiol. 2020;76(16):1878–901. 33059834 10.1016/j.jacc.2020.08.042 PMC7808240

[2] Lyle MA, Cooper LT Jr. Cardiovascular Outcomes in Sarcoidosis. J Am Coll Cardiol. 2020;76(7):778–80. 32792074 10.1016/j.jacc.2020.06.046

[3] Longcope WT, Freiman DG. A study of sarcoidosis; based on a combined investigation of 160 cases including 30 autopsies from The Johns Hopkins Hospital and Massachusetts General Hospital. Medicine (Baltimore). 1952;31(1):1–132. 14899212

[4] Silverman KJ, Hutchins GM, Bulkley BH. Cardiac sarcoid: a clinicopathologic study of 84 unselected patients with systemic sarcoidosis. Circulation. 1978;58(6):1204–11. 709777 10.1161/01.cir.58.6.1204

[5] Sharma OP, Maheshwari A, Thaker K. Myocardial sarcoidosis. Chest. 1993;103(1):253–8. 8417889 10.1378/chest.103.1.253

[6] Matsui Y, et al. Clinicopathological study of fatal myocardial sarcoidosis. Ann N Y Acad Sci. 1976;278:455–69. 1067031 10.1111/j.1749-6632.1976.tb47058.x

[7] Cooper LT, et al. The role of endomyocardial biopsy in the management of cardiovascular disease: a scientific statement from the American Heart Association, the American College of Cardiology, and the European Society of Cardiology Endorsed by the Heart Failure Society of America and the Heart Failure Association of the European Society of Cardiology. Eur Heart J. 2007;28(24):3076–93. 17959624 10.1093/eurheartj/ehm456

[8] Kolluri N, et al. Routine Laboratory Biomarkers As Prognostic Indicators of Cardiac Sarcoidosis Outcomes. Sarcoidosis Vasc Diffuse Lung Dis. 2022;39(3):e2022023. 36791034 10.36141/svdld.v39i2.11136 PMC9766851

[9] Ning N, et al. Serial Cardiac FDG-PET for the Diagnosis and Therapeutic Guidance of Patients With Cardiac Sarcoidosis. J Card Fail. 2019;25(4):307–11. 30825644 10.1016/j.cardfail.2019.02.018

[10] Birnie DH, et al. HRS expert consensus statement on the diagnosis and management of arrhythmias associated with cardiac sarcoidosis. Heart Rhythm. 2014;11(7):1305–23. 24819193 10.1016/j.hrthm.2014.03.043

[11] Feldman AM, McNamara D. Myocarditis. N Engl J Med. 2000;343(19):1388–98. 11070105 10.1056/NEJM200011093431908

[12] Trachtenberg BH, Hare JM. Inflammatory Cardiomyopathic Syndromes. Circ Res. 2017;121(7):803–18. 28912184 10.1161/CIRCRESAHA.117.310221

[13] Fung G, et al. Myocarditis. Circ Res. 2016;118(3):496–514. 26846643 10.1161/CIRCRESAHA.115.306573

[14] Fu M, et al. Trends in myocarditis incidence, complications and mortality in Sweden from 2000 to 2014. Sci Rep. 2022;12(1):1810. 35110692 10.1038/s41598-022-05951-z PMC8810766

[15] Sagar S, Liu PP, Cooper LT Jr. Myocarditis. Lancet. 2012;379(9817):738–47. 22185868 10.1016/S0140-6736(11)60648-X PMC5814111

[16] Tschope C, et al. Myocarditis and inflammatory cardiomyopathy: current evidence and future directions. Nat Rev Cardiol. 2021;18(3):169–93. 33046850 10.1038/s41569-020-00435-x PMC7548534

[17] Crisci G, Bobbio E, Gentile P, Bromage DI, Bollano E, Ferone E, ..., Salzano, A. Biomarkers in acute myocarditis and chronic inflammatory cardiomyopathy: an updated review of the literature. J Clin Med. 2023;12(23):7214. 10.3390/jcm12237214 PMC10706911 38068265

[18] Maisch B. Cardio-Immunology of Myocarditis: Focus on Immune Mechanisms and Treatment Options. Front Cardiovasc Med. 2019;6:48. 31032264 10.3389/fcvm.2019.00048 PMC6473396

[19] Ammirati E, et al. Management of Acute Myocarditis and Chronic Inflammatory Cardiomyopathy: An Expert Consensus Document. Circ Heart Fail. 2020;13(11):e007405. 33176455 10.1161/CIRCHEARTFAILURE.120.007405 PMC7673642

[20] Suresh A, Martens P, Tang WHW. Biomarkers for Myocarditis and Inflammatory Cardiomyopathy. Curr Heart Fail Rep. 2022;19(5):346–55. 35913661 10.1007/s11897-022-00569-8 PMC9340754

[21] Biomarkers Definitions Working G. Biomarkers and surrogate endpoints: preferred definitions and conceptual framework. Clin Pharmacol Ther. 2001;69(3):89–95. 11240971 10.1067/mcp.2001.113989

[22] Gungor S, et al. Conventional markers in determination of activity of sarcoidosis. Int Immunopharmacol. 2015;25(1):174–9. 25623898 10.1016/j.intimp.2015.01.015

[23] Ungprasert P, et al. Diagnostic Utility of Angiotensin-Converting Enzyme in Sarcoidosis: A Population-Based Study. Lung. 2016;194(1):91–5. 26563332 10.1007/s00408-015-9826-3 PMC4768304

[24] Casanova N, et al. Identifying Novel Biomarkers in Sarcoidosis Using Genome-Based Approaches. Clin Chest Med. 2015;36(4):621–30. 26593137 10.1016/j.ccm.2015.08.005 PMC4990072

[25] Kiko T, et al. A Multiple Biomarker Approach in Patients with Cardiac Sarcoidosis. Int Heart J. 2018;59(5):996–1001. 30101857 10.1536/ihj.17-695

[26] Hulten E, et al. Presence of Late Gadolinium Enhancement by Cardiac Magnetic Resonance Among Patients With Suspected Cardiac Sarcoidosis Is Associated With Adverse Cardiovascular Prognosis: A Systematic Review and Meta-Analysis. Circ Cardiovasc Imaging. 2016;9(9):e005001. 27621357 10.1161/CIRCIMAGING.116.005001 PMC5449111

[27] Ise T, et al. Extensive late gadolinium enhancement on cardiovascular magnetic resonance predicts adverse outcomes and lack of improvement in LV function after steroid therapy in cardiac sarcoidosis. Heart. 2014;100(15):1165–72. 24829369 10.1136/heartjnl-2013-305187

[28] Rothkrantz-Kos S, et al. Potential usefulness of inflammatory markers to monitor respiratory functional impairment in sarcoidosis. Clin Chem. 2003;49(9):1510–7. 12928233 10.1373/49.9.1510

[29] Eurelings LEM, et al. Sensitivity and specificity of serum soluble interleukin-2 receptor for diagnosing sarcoidosis in a population of patients suspected of sarcoidosis. PLoS ONE. 2019;14(10):e0223897. 31622413 10.1371/journal.pone.0223897 PMC6797090

[30] Kobayashi Y, et al. Association of high serum soluble interleukin 2 receptor levels with risk of adverse events in cardiac sarcoidosis. ESC Heart Fail. 2021;8(6):5282–92. 34514715 10.1002/ehf2.13614 PMC8712796

[31] Kandolin R, et al. Usefulness of Cardiac Troponins as Markers of Early Treatment Response in Cardiac Sarcoidosis. Am J Cardiol. 2015;116(6):960–4. 26209113 10.1016/j.amjcard.2015.06.021

[32] Tsutsui H, et al. Natriuretic Peptides: Role in the Diagnosis and Management of Heart Failure: A Scientific Statement From the Heart Failure Association of the European Society of Cardiology, Heart Failure Society of America and Japanese Heart Failure Society. J Card Fail. 2023;29(5):787–804. 37117140 10.1016/j.cardfail.2023.02.009

[33] Handa T, et al. Significance of plasma NT-proBNP levels as a biomarker in the assessment of cardiac involvement and pulmonary hypertension in patients with sarcoidosis. Sarcoidosis Vasc Diffuse Lung Dis. 2010;27(1):27–35. 21086902

[34] Yasutake H, et al. Detection of cardiac sarcoidosis using cardiac markers and myocardial integrated backscatter. Int J Cardiol. 2005;102(2):259–68. 15982494 10.1016/j.ijcard.2004.05.028

[35] Yancy CW, et al. 2013 ACCF/AHA guideline for the management of heart failure: a report of the American College of Cardiology Foundation/American Heart Association Task Force on Practice Guidelines. J Am Coll Cardiol. 2013;62(16):e147-239. 23747642 10.1016/j.jacc.2013.05.019

[36] Ponikowski P, et al. 2016 ESC Guidelines for the diagnosis and treatment of acute and chronic heart failure. Kardiol Pol. 2016;74(10):1037–147. 27748494 10.5603/KP.2016.0141

[37] Miyakuni S, et al. The Prognostic Value of B-Type Natriuretic Peptide in Patients With Cardiac Sarcoidosis Without Heart Failure: Insights From ILLUMINATE-CS. J Am Heart Assoc. 2022;11(24): e025803. 10.1161/JAHA.122.025803 PMC9798822 36515231

[38] Filipovic S, Violeta V, Jelica V, Mihailo S, Aleksandar J. Vitamin D deficiency and activity of sarcoidosis. Eur Respir J. 2016.

[39] Ide T, et al. Mitochondrial DNA damage and dysfunction associated with oxidative stress in failing hearts after myocardial infarction. Circ Res. 2001;88(5):529–35. 11249877 10.1161/01.res.88.5.529

[40] Suematsu N, et al. Oxidative stress mediates tumor necrosis factor-alpha-induced mitochondrial DNA damage and dysfunction in cardiac myocytes. Circulation. 2003;107(10):1418–23. 12642364 10.1161/01.cir.0000055318.09997.1f

[41] Kobayashi S, et al. Urinary 8-hydroxy-2’-deoxyguanosine reflects symptomatic status and severity of systolic dysfunction in patients with chronic heart failure. Eur J Heart Fail. 2011;13(1):29–36. 20965876 10.1093/eurjhf/hfq178

[42] Susa T, et al. Urinary 8-hydroxy-2’-deoxyguanosine as a novel biomarker for predicting cardiac events and evaluating the effectiveness of carvedilol treatment in patients with chronic systolic heart failure. Circ J. 2012;76(1):117–26. 22008315 10.1253/circj.cj-11-0537

[43] Ishiguchi H, Kobayashi S, Myoren T, Kohno M, Nanno T, Murakami W, ..., Yano M. Urinary 8-hydroxy-2′-deoxyguanosine as a myocardial oxidative stress marker is associated with ventricular tachycardia in patients with active cardiac sarcoidosis. Circ Cardiovasc Imaging. 2017;10(12):e006764. 10.1161/CIRCIMAGING.117.006764 29208596

[44] Myoren T, et al. An oxidative stress biomarker, urinary 8-hydroxy-2’-deoxyguanosine, predicts cardiovascular-related death after steroid therapy for patients with active cardiac sarcoidosis. Int J Cardiol. 2016;212:206–13. 27043062 10.1016/j.ijcard.2016.03.003

[45] Kobayashi S, et al. Urinary 8-hydroxy-2’-deoxyguanosine as a novel biomarker of inflammatory activity in patients with cardiac sarcoidosis. Int J Cardiol. 2015;190:319–28. 25935620 10.1016/j.ijcard.2015.04.144

[46] Ambros V. The functions of animal microRNAs. Nature. 2004;431(7006):350–5. 15372042 10.1038/nature02871

[47] Iorio MV, Croce CM. MicroRNAs in cancer: small molecules with a huge impact. J Clin Oncol. 2009;27(34):5848–56. 19884536 10.1200/JCO.2009.24.0317 PMC2793003

[48] Wang HW, et al. Deficiency of the microRNA-31-microRNA-720 pathway in the plasma and endothelial progenitor cells from patients with coronary artery disease. Arterioscler Thromb Vasc Biol. 2014;34(4):857–69. 24558106 10.1161/ATVBAHA.113.303001

[49] Fujiwara W, et al. Serum microRNA-126 and -223 as new-generation biomarkers for sarcoidosis in patients with heart failure. J Cardiol. 2018;72(6):452–7. 30054123 10.1016/j.jjcc.2018.06.004

[50] Agbor-Enoh S, et al. Cell-Free DNA to Detect Heart Allograft Acute Rejection. Circulation. 2021;143(12):1184–97. 33435695 10.1161/CIRCULATIONAHA.120.049098 PMC8221834

[51] University of Iowa. Cell free DNA in cardiac sarcoidosis. Myocarditis foundation. Cell free DNA in cardiac sarcoidosis. n.d. https://www.Myocarditisfoundation.org/Clinical-Trial/Cell-Free-Dna-In-Cardiac-Sarcoidosis/ .

[52] Rose NR. Critical cytokine pathways to cardiac inflammation. J Interferon Cytokine Res. 2011;31(10):705–10. 21861699 10.1089/jir.2011.0057 PMC3189548

[53] Hua X, et al. Single-Cell RNA Sequencing to Dissect the Immunological Network of Autoimmune Myocarditis. Circulation. 2020;142(4):384–400. 32431172 10.1161/CIRCULATIONAHA.119.043545

[54] Cai Y, et al. Heparin-Binding Protein: A Novel Biomarker Linking Four Different Cardiovascular Diseases. Cardiol Res Pract. 2020;2020:9575373. 32612856 10.1155/2020/9575373 PMC7312699

[55] Muller I, et al. Serum alarmin S100A8/S100A9 levels and its potential role as biomarker in myocarditis. ESC Heart Fail. 2020;7(4):1442–51. 32462801 10.1002/ehf2.12760 PMC7373886

[56] Smith SC, et al. Elevations of cardiac troponin I associated with myocarditis Experimental and clinical correlates. Circulation. 1997;95(1):163–8. 8994432

[57] Caforio AL, et al. Current state of knowledge on aetiology, diagnosis, management, and therapy of myocarditis: a position statement of the European Society of Cardiology Working Group on Myocardial and Pericardial Diseases. Eur Heart J. 2013;34(33):2636–48. 23824828 10.1093/eurheartj/eht210

[58] Palaskas N, et al. Immune Checkpoint Inhibitor Myocarditis: Pathophysiological Characteristics, Diagnosis, and Treatment. J Am Heart Assoc. 2020;9(2):e013757. 31960755 10.1161/JAHA.119.013757 PMC7033840

[59] Mahmood SS, et al. Myocarditis in Patients Treated With Immune Checkpoint Inhibitors. J Am Coll Cardiol. 2018;71(16):1755–64. 29567210 10.1016/j.jacc.2018.02.037 PMC6196725

[60] Brahmer JR, Lacchetti C, Thompson JA. Management of Immune-Related Adverse Events in Patients Treated With Immune Checkpoint Inhibitor Therapy: American Society of Clinical Oncology Clinical Practice Guideline Summary. J Oncol Pract. 2018;14(4):247–9. 29517954 10.1200/JOP.18.00005

[61] Jensen J, et al. Inflammation increases NT-proBNP and the NT-proBNP/BNP ratio. Clin Res Cardiol. 2010;99(7):445–52. 20229122 10.1007/s00392-010-0140-z

[62] Nie X, et al. Circulating miR-4763-3p Is a Novel Potential Biomarker Candidate for Human Adult Fulminant Myocarditis. Mol Ther Methods Clin Dev. 2020;17:1079–87. 32478123 10.1016/j.omtm.2020.05.005 PMC7248292

[63] Blanco-Dominguez R, et al. A Novel Circulating MicroRNA for the Detection of Acute Myocarditis. N Engl J Med. 2021;384(21):2014–27. 34042389 10.1056/NEJMoa2003608 PMC8258773

[64] Zhang Y, et al. Serum exosome microRNA panel as a noninvasive biomarker for molecular diagnosis of fulminant myocarditis. Mol Ther Methods Clin Dev. 2021;20:142–51. 33473354 10.1016/j.omtm.2020.11.006 PMC7786026

[65] Shibata N, et al. Clinical Value of Troponin Levels to Cardiac Function and Prognosis in Patients with Fulminant Myocarditis. Int Heart J. 2024;65(2):218–29. 38556333 10.1536/ihj.23-589

[66] Wang J, et al. Soluble ST2 Is a Sensitive and Specific Biomarker for Fulminant Myocarditis. J Am Heart Assoc. 2022;11(7):e024417. 35377184 10.1161/JAHA.121.024417 PMC9075487

[67] Cheng CY, et al. Myocarditis in systemic immune-mediated diseases: Prevalence, characteristics and prognosis A systematic review. Autoimmun Rev. 2022;21(4):103037. 34995763 10.1016/j.autrev.2022.103037

[68] Kaya Z, Leib C, Katus HA. Autoantibodies in heart failure and cardiac dysfunction. Circ Res. 2012;110(1):145–58. 22223211 10.1161/CIRCRESAHA.111.243360

[69] Matsumori A, et al. Immunoglobulin free light chains: an inflammatory biomarker of diabetes. Inflamm Res. 2020;69(8):715–8. 32424470 10.1007/s00011-020-01357-7

[70] Bang V, et al. Management of Patients With Giant Cell Myocarditis: JACC Review Topic of the Week. J Am Coll Cardiol. 2021;77(8):1122–34. 33632487 10.1016/j.jacc.2020.11.074

[71] Gilotra NA, et al. Lack of Relationship Between Serum Cardiac Troponin I Level and Giant Cell Myocarditis Diagnosis and Outcomes. J Card Fail. 2016;22(7):583–5. 26768222 10.1016/j.cardfail.2015.12.022

[72] Ekstrom K, et al. Long-term outcome and its predictors in giant cell myocarditis. Eur J Heart Fail. 2016;18(12):1452–8. 27407025 10.1002/ejhf.606

[73] Ammirati E, et al. Acute and Fulminant Myocarditis: a Pragmatic Clinical Approach to Diagnosis and Treatment. Curr Cardiol Rep. 2018;20(11):114. 30259175 10.1007/s11886-018-1054-z

[74] Al Ali AM, et al. Eosinophilic myocarditis: case series and review of literature. Can J Cardiol. 2006;22(14):1233–7. 17151774 10.1016/s0828-282x(06)70965-5 PMC2569073

[75] Brambatti M, et al. Eosinophilic Myocarditis: Characteristics, Treatment, and Outcomes. J Am Coll Cardiol. 2017;70(19):2363–75. 29096807 10.1016/j.jacc.2017.09.023

[76] Pieroni M, et al. Clozapine-induced hypersensitivity myocarditis. Chest. 2004;126(5):1703–5. 15539749 10.1378/chest.126.5.1703

[77] Ammirati E, et al. A life-threatening presentation of eosinophilic granulomatosis with polyangiitis. J Cardiovasc Med (Hagerstown). 2016;17(Suppl 2):e109–11. 26556445 10.2459/JCM.0000000000000330

[78] Zhong Z, et al. Diagnosis and treatment of eosinophilic myocarditis. J Transl Autoimmun. 2021;4:100118. 35005589 10.1016/j.jtauto.2021.100118 PMC8716607

[79] Seguela PE, et al. Eosinophilic cardiac disease: Molecular, clinical and imaging aspects. Arch Cardiovasc Dis. 2015;108(4):258–68. 25858537 10.1016/j.acvd.2015.01.006

[80] Arima M, Kanoh T. Eosinophilic myocarditis associated with dense deposits of eosinophil cationic protein (ECP) in endomyocardium with high serum ECP. Heart. 1999;81(6):669–71. 10336931 10.1136/hrt.81.6.669 PMC1729068

[81] Arima M, et al. Serum levels of eosinophil cationic protein in patients with eosinophilic myocarditis. Int J Cardiol. 2002;84(1):97–9. 12104073 10.1016/s0167-5273(02)00074-8

